# Plasma *GBP2* promoter methylation is associated with advanced stages in breast cancer

**DOI:** 10.1590/1678-4685-GMB-2019-0230

**Published:** 2020-11-17

**Authors:** Farzaneh Rahvar, Mahdieh Salimi, Hossein Mozdarani

**Affiliations:** 1 National Institute of Genetic Engineering and Biotechnology (NIGEB), Institute of Medical Biotechnology, Department of Medical Genetics, Tehran, Iran.; 2 Tarbiat Modares University, Faculty of Medical Sciences, Department of Medical Genetics, Tehran, Iran.

**Keywords:** Breast cancer, biomarker, cell-free nucleic acids, methylation, GBP2

## Abstract

Blood methylated cell-free DNA (cfDNA) as a minimally invasive cancer biomarker has great importance in cancer management. Guanylate binding protein 2 (GBP2) has been considered as a possible controlling factor in tumor development. *GBP2* gene expression and its promoter methylation status in both plasma cfDNA and tumor tissues of ductal carcinoma breast cancer patients were analyzed using SYBR green comparative Real-Time RT-PCR and, Methyl-specific PCR techniques, respectively in order to find a possible cancer-related marker. The results revealed that *GBP2* gene expression and promoter methylation were inversely associated. *GBP2* was down-regulated in tumors with emphasis on triple negative status, nodal involvement and higher cancer stages (*p*<0.0001). *GBP2* promoter methylation on both cfDNA and tumor tissues were positively correlated and was detected in about 88% of breast cancer patients mostly in (Lymph node positive) LN+ and higher stages. Data provided shreds of evidence that *GBP2* promoter methylation in circulating DNA may be considered as a possible effective non-invasive molecular marker in poor prognostic breast cancer patients with the evidence of its relation to disease stage and lymph node metastasis. However further studies need to evaluate the involvement of *GBP2* promoter methylation in progression-free survival or overall survival of the patients.

## Introduction

Breast cancer is regarded globally (Siegel *et al.*, 2013) as the most common cause of death and malignancy in females (Asiaf *et al.*, 2014). Despite considerable signs of progress have been made in breast cancer diagnosis and therapies, unfortunately, the incidence rate is still high. Early detection may promote successful treatment and outcomes. Therefore recognizing cancer molecular mechanisms might lead to finding new biomarkers with diagnostic, prognostic or therapeutic importance.

Epigenetic changes are considered as cancer hallmarks and may be associated with tumor development (Wu *et al.*, 2015). CpG islands hyper-methylation in the promoter areas might have an impact on inactivation of important elements such as the tumor suppressor genes, DNA repair genes, cell cycle regulators and transcription factors (Wu *et al.*, 2015). The analysis of aberrant methylation in the promoting region of efficient genes can be extremely significant in the early identification and risk assessment of precursor lesions as a result of participation in cancer initiation and advancement (Nakamura *et al.*, 2014; Semaan et al, 2016; Stearns *et al.* 2016; Terry *et al.*, 2016; Hao *et al.*, 2017). Also, the dynamic nature of DNA methylation events makes them promising as therapeutic targets in cancer management strategies (Szarc *et al.*, 2017).

Early diagnosis and/or prognosis biomarkers based on analyses of extra cellular nucleic acid in organic liquids were attempted to create non-invasive approaches for the detection of cancer. Liquid biopsy is a potential alternative for tissue biopsy. DNA can release from normal and cancerous apoptotic cells into the circulating blood system. There is enormous potential with the use of circulating DNA to provide a non-invasive personalized genomic snapshot of the tumor of a patient. Although the enhanced DNA concentration itself in the circulation of plasma could be regarded as an indicator of growth but is not sufficiently particular, in other diseases such as rheumatoid arthritis, systemic lupus erythematosus, pancreas, glomerulonephritis and hepatitis, where the similar rise of plasma DNA concentration also occurred (Han *et al.* 2017). So, finding the informative biomarkers expressing tumors in circulating blood may have great importance in cancer management. Epigenetic alternations, such as gene promoter methylation may be considered as novel cancer biomarkers with prognostic, diagnostic or predictive value in different stages of the variety of cancers (Chen *et al.*, 2017). The various malignancies have been recorded for tumor-specific changes, such as aberrant promoter methylation in circulating plasma or serum-based DNA of patients. DNA methylation changes are such a frequent molecular change, including breast cancer (Rauscher *et al.*, 2015), in human neoplasia (Cho *et al.*, 2010). The epigenetic changes can lead to neoplastic process through transcriptional silencing of tumor suppressor genes or inducing oncogenes and may lead to initial steps of tumor cell proliferation induction (Cho *et al.*, 2010). Therefore, assessment of the pattern of methylation in the early detection of cancer could be regarded with considerable importance. This field of study has acquired enormous momentum in the last few years. Several studies have demonstrated the potential for circulating DNA to predict prognosis and treatment response in metastatic breast cancer. The exosomal miRNA and plasma hyper-methylated DNA were shown to be promising for early breast cancer identification and could be used as indicators for therapeutic reactions (Beddowes *et al.*, 2017).

Guanylate-binding protein (GBP) belongs to the superfamily of INF-inducible guanosine triphosphate hydrolases (GTPases). Up to now, seven human GBP genes, including guanylate-binding protein 1 -7 have been reported. GBPs, such as GBP1 and GBP2, have antimicrobial and antiviral activities in host defense and may act as protective factors in host defense, autoimmunity and controlling infection (Wang *et al.*, 2018). The roles of GBP genes in cancers are complicated. Some GBP family members were expressed in variety of cancers such as breast cancer, colorectal cancer (CRC), oral squamous cell carcinoma (OSCC), esophageal squamous cell carcinomas (SCC), prostate cancer, cutaneous T-cell lymphoma, and Kaposi's sarcoma (Wang *et al.*, 2018). As a possible regulatory factor in the development of tumors, guanylate binding protein 2 (GBP2) attracted attention (Guimaraes *et al.*, 2009; Kobayashi *et al.*, 2010; Messmer-Blust *et al.*, 2010; Balasubramanian *et al.*, 2011). GBP2 inhibits Rac and NF-kappa B proteins and consequently acts against matrix metallopeptidase 9 (MMP9) activation (Balasubramanian *et al.*, 2011). 

It was reported that GBP2 interacts with dynamin-related protein 1 (Drp1) and blocks translocation of Drp1 to mitochondria, thereby attenuating Drp1-dependent mitochondrial fission and invasion of breast cancer cells and may represent a new therapeutic target to suppress breast cancer metastasis through attenuation of Drp1-dependent mitochondrial fission (Zhang *et al.*, 2017). 

Considering the important role of GBP2 as a possible tumor suppressor gene and the increasing importance of epigenetics in gene regulations and cancer management, GBP2 expression in breast cancer tumors with different histo-clinical characteristics and for the first time, its promoter methylation as a possible epigenetic factor that regulates this gene expression, were studied in both plasma and tumors.

## Material and Methods

### Subjects

The research was performed as a case/control study. The test samples were categorized into two groups, breast tissues, and plasma. The normal adjacent, tumor breast tissues, and 10 mL peripheral blood of 84 patients admitted to Imam-Khomeini Hospital (Tehran, Iran) between the years 2012 to 2016, were recruited before initiation of any therapy. Furthermore, 20 normal breast tissues from females intended to breast reduction surgery with cosmetic purpose and without any malignancy history were considered as the control group. The 75 unaffected female blood donors were enrolled as the blood control group who did not have any breast lesion neither in them nor their first-degree relatives. The ethical committee of the National Institute of Genetic Engineering and Biotechnology (NIGEB) approved the protocol based on the Helsinki declaration. Patients and controls signed a written informed consent letter before enrolment. 

The inclusion criteria for the patient samples were the histopathological diagnosis of ductal carcinoma and availability of immunohistochemistry (IHC) results for human epidermal growth factor 2 (HER-2), estrogen receptors (ER) and progesterone receptor (PR) status and other pathologic diagnostic information as well as good quality of extracted RNA and DNA . Chemotherapy or radiation therapy prior to recruitment and any history of family breast disease or malignancy were regarded as exclusion criteria.

The patients were divided into three groups according to the level of the tumor (stage II to IV). Details of the patient clinicopathological parameters are presented in Table 1.

**Table 1 -:** Demographic and histoclinical characteristics of patients and normal controls.

	Patient N (%)	Control N (%)	Normal control (breast reduction)
number	84	75	20
Age (years)			
Mean	47.2±12.6	48.5±16.4	37±10.2
Range	27-84	25-80	24-57
Stage at diagnosis			
Stage II	43 (51.2%)		
Stage III	28 (33.3%)		
Stage IV	13 (15.5%)		
Lymph node status			
N0	35 (41.7%)		
N+	49(58.3%)		
Distance metastasis			
Yes	14 [3 bone, 11 lung] (16.7%)		
No	70 (83.3%)		
Receptor status (IHC)			
ER-positive	50 (59.5%)		
ER-negative	34 (40.5%)		
PR-positive	45 (53.6%)		
PR-negative	39 (46.4%)		
HER2 +	23 (27.4%)		
HER2 -	52(61.9%)		
TNBC	9 (10.7%)		
Non-TNBC	75(98.3)		
Menopause status			
Yes	45 (53.6%)	36 (48%)	
No	39(46.4%)	39 (52%)	
Smoking			
Yes	21 (25%)	24 (32%)	
No	63 (75%)	51 (68%)	
Pregnancy at term			
Yes	69 (82.1%)	60(80%)	
No	15 (17.9%)	15 (20%)	
HRT			
Yes	19 (22.6%)	15 (20%)	
No	65 (77.4%)	60 (80%)	

_ER: Estrogen Receptor, PR: Progesterone Receptor, HER2: Human Epidermal Growth Factor Receptor 2, HRT: Hormone Replacement Therapy, Triple Negative: TN._

### Sample collection

Tissues were immediately snap-frozen and stored at -80 °C within 2 h. Totally 188 breast tissue specimens were collected as 84 ductal carcinoma breast tumors, 84 normal tissues near the tumor region, named normal adjacent, and 20 normal control breast tissues. 

One hundred and fifty-nine blood samples, comprised of 84 breast cancer patients and 75 normal unaffected controls, were collected before surgery. Peripheral blood (10 mL in ethylene diamine tetra acetic acid (EDTA)) was obtained in the middle of vein puncture after the first 2 mL of blood was discarded.

### RNA extraction and cDNA synthesis

Total RNA was extracted from tissues; using RNX plus Extraction Kit (CinnaGen Co., Iran), RNX-Plus is a guanidine/phenol solution for total RNA isolation from the homogenized sample. Through the action of guanidine salt in RNA isolation procedure, at the same time, DNA and protein were precipitated in the phenol phase. The aqueous phase contains high quality and all types of the genomic RNA. Two micrograms of total RNA was digested by 2 µg DNase 1 (Fermentas, Manchester, UK) to remove genomic DNA contamination and then 1 µg of RNA was used for cDNA synthesis, with Precision qScript^T^ Reverse Transcription Kit (Primerdesign, Chandlers’s Ford, UK). All the steps were done according to the manufacturer’s instructions.

### DNA extraction from tissue and plasma

DNA was extracted from tissues in accordance with the protocol of the manufacturer using a DNeasy Blood and Tissue kit (Qiagen, Hiden, Germany).

Freshly collected blood was processed by a 1-hour centrifugation at 1000 × *g* for 10 minutes at 4° C. Without disturbing the cellular layer the supernatant was carefully transferred into a Falcon tube and centrifuged for 10 min to remove any remaining cells. Cell-free plasma was then aliquoted and stored at -80 °C. DNA was extracted from a 0.5 ml plasma aliquot with QIAmp DNA Blood Midi Kit (Qiagen, Hiden, Germany) according to the manufacturer’s instructions and stored at -20 °C before further analysis. We have already reported the quality and integrity of plasma cfDNA in breast cancer patients compared with control (Salimi *et al.* 2019).

### Standard curve construction

**Figure 1 -:**
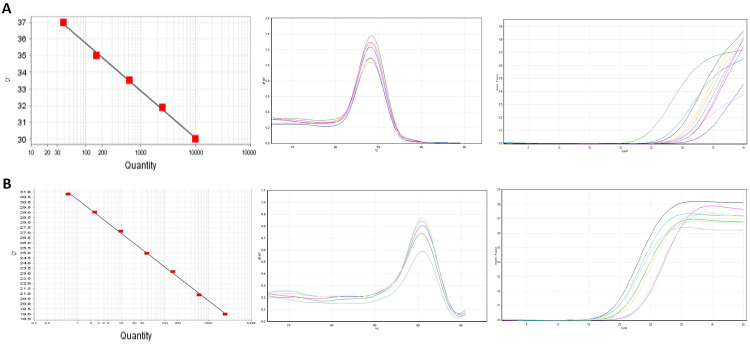
Standard curve, melt curve and amplification plot of *GBP2* (A) and *beta-actin* (B) genes. Standard curves showed good linearity and amplification efficiency (100-101%) for each primer set of experimental and reference (beta-actin) gene.

Amplification efficiency for each primer pair was determined by the amplification of a linear standard curve (from 0.24 to 1,000 ng) of total cDNA assessed by ultraviolet spectrophotometry. For the primer set of experimental (*GBP2*) and reference (*beta-actin*) genes, standard curves were shown to have good linearity and amplification (100%) (Figure 1).

### Real-time RT-PCR analysis

A Light Cycling TM system (Rotor, Corbett, Germany) was used for all Real-time RT-PCRs. For each sample, 500 ng/µl of total cDNA was used. cDNA was mixed with 0.3 µM of each forward and reverse primers with 10 µL of SYBR green master mix (Roche, Germany) to a ﬁnal reaction volume of 20 µl. The forward and reverse primer sequences were

F: 5′CATCACTCCTGCCAAGTGGT3′, 

R: 5′ACAGATCATGCAGCCTCCAC3 and 

F: 5 GAGACCTTCAACACCCCAGC3′

R: 5′ AGACGCAGGATGGCATGG 3′ for *GBP2* and *beta-actin* genes respectively.

The thermal cycling conditions comprised of an initial denaturation step at 95 °C for 7 min and 45 cycles at 95 °C for 10 s and 61 °C for 30 s and 72 °C for 20 s. Experiments were performed with triplicates for each data point. Using the 2^-ΔΔCT^ method (Livak and Schmittgen, 2001) the data were presented as the fold change in gene expression normalized to an endogenous reference gene (*beta-actin*) and relative to the controls. The two-fold and more RNA expression considered as up-regulation, between 0.5- and 2-fold as normal and 0.5-fold and less as down-regulation. The melt and amplification cures of each primer are depicted in Figure 1.

### Methylation-specific PCR (MS-PCR) for evaluation of gene methylation status

**Figure 2 -:**
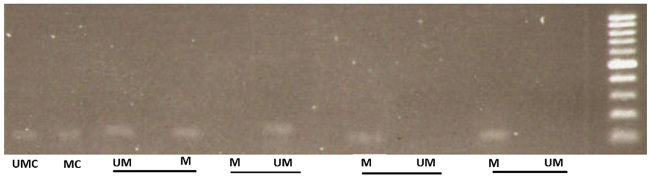
The *GBP2* methylation pattern statues in 1.5% agarose gel post electrophoresis. The molecular marker size is 100bp. M: methylated, UM: unmethylated, MC: methylated control, UMC: unmethylated control.

Bisulfite isolated DNA treatment was performed according to the manufacturer's instructions, using the EpiTect Bisulfite Kit (Qiagen, Hilden, Germany). The methylation status of the *GBP2* gene was determined qualitatively by the methylation-specific polymerase chain reaction (MS-PCR). MS-PCR primer sequences designed for a CPG island in the promoter region of *GBP2* gene, are listed as follow: *GBP2* methylated-specific F: 5’- TGGAGGAAGTTTTAGGACGT-3’ and R: 5’- CTCCTCTCTTTTCTTCCGAA-3’. The unmethylated *GBP2* primers: 

5’-GTTGGAGGAAGTTTTAGGATGT-3’ and 5’- TCCTCCTCTCTTTTCTTCCAAA-3’. The amplicon size and annealing temperature were 108 base pair and 56 °C, respectively. Four microliters of bisulfite-modified DNA was subjected to PCR amplification in a final reaction volume of 25 𝜇L comprised of 12.5 𝜇l of 2x EpiTect MSP Kit (Qiagen, Hilden, Germany) and 0.5 𝜇M of each primer. PCR was performed with an initial 10 minutes incubation at 95 °C, followed by 40 cycles of denaturation at 95 °C for 30 s, annealing at 56 °C for 30 s, extension at 72 °C for 60 s, and a final 10 min hold at 72 °C. Each sample was assessed in duplicate, and each run included no template control (NTC) and external universal control (methylated and unmethylated DNA) using the EpiTek PCR control DNA set (Qiagen, Hilden, Germany). On 1.5% agarose gels, the aliquots of PCR products were separated. The gels were stained with ethidium bromide and photographed under UV illumination. For each sample two MS-PCR were performed using methylated and unmethylated primers. The *GBP2* methylation pattern statues in agarose gel post electrophoresis were shown in Figure 2. 

### Statistical analysis

Graphpad Prism 8.0.2 (California Corporation, USA) was used to analyze the data. The Mann-Whitney U test and Kruskal-Wallis test were performed for numerical data and the Chi-square test was used to analyze the relationship between parameter data. Numerical data are presented as the mean ± standard deviation (SD). Differences were considered as statistically significant if 𝑝 <0.05.

## Results

### *GBP2* expression in tumor and normal breast tissues 

**Figure 3 -:**
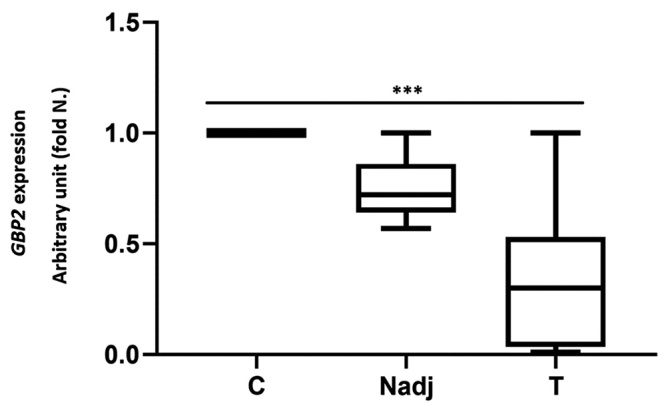
The Real-time RT-PCR analysis of *GBP2* expression in ductal carcinoma and adjacent normal and normal control of breast tissues. Results are expressed as fold number changed versus control assumed as 1. *GBP2* RNA values were previously normalized to *beta-actin* values. Tumor (T) compared with normal control (C) and Normal adjacent (Nadj) groups using Kruskal-Wallis test, ***: *p*<0.0001.

As shown in Figure 3, results indicated that the overall expression of *GBP2* was significantly down-regulated in breast tumors compared with normal adjacent tissues and normal control (*p*<0.0001). The mean of *GBP2* expression in ductal carcinoma breast tumors was 0.31± 0.28 fold change with the range of 0.01 -1 and 0.26 ± 0.26 fold change with the range of 0.01 to 0.93 compared with normal adjacent tissues and normal control, respectively. In tumor tissues, the frequency of *GBP2* down regulation was 73.2% whereas 26.8% of breast cancer patients showed normal expression and none of the tested samples showed *GBP2* up-regulation.

### *GBP2* expression in different breast cancer groups based on histopathology situations 

**Figure 4 -:**
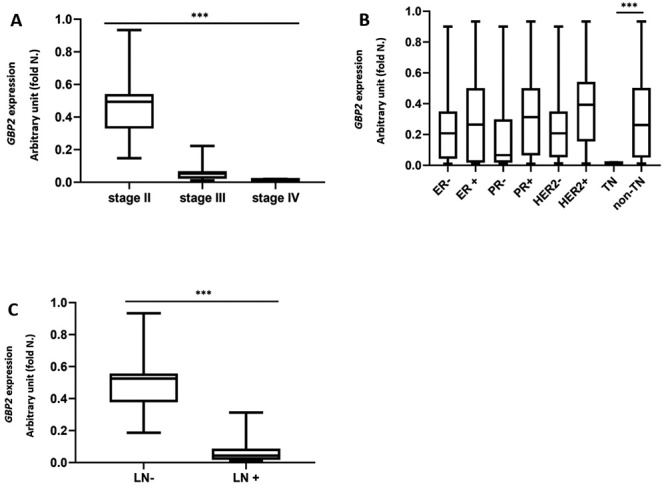
Mean of *GBP2* expression in breast cancer tumors stratified according to: A) stages B) hormone receptor status and C) lymph node involvement situations. ER: estrogen receptor, PR: progesterone receptor, HER2: human epidermal growth factor receptor, LN: Lymph node, TN: triple negative (ER-, PR-, HER2-). ***: *p*<0.0001. Statistical method: Kruskal-Wallis test (A), Mann-Whitney test (B & C).

As shown in Figure 4, the data demonstrated that the level of *GBP2* was significantly reduced in triple negative (ER-, PR-, HER2-), higher stages of the disease and LN+ tumors compared with their non-triple negative, lower stages, and LN- counterparts (*p*<0.001). The *GBP2* expression value was not significantly different in different hormone receptor statues comprised of having or not having estrogen, progesterone and human epidermal growth factor receptor 2.

### *GBP2* promoter methylation status in breast tissues and plasma 

A region of *GBP2* promoter methylation status in plasma and breast tissues of breast cancer patients compared with normal control were studied. There were three patterns of the studied region of *GBP2* promoter methylation, they were 1) methylated, 2) unmethylated and 3) both methylated and unmethylated patterns (Figure 2).

**Figure 5 -:**
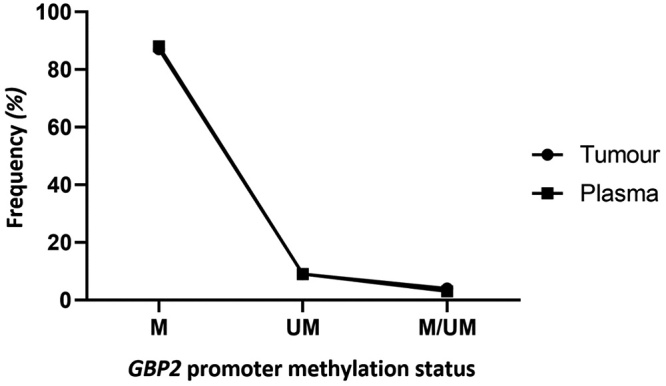
Frequency matching of *GBP2* promoter methylation percentage in tumor and plasma samples of breast cancer patients. M: Methylated promoter; UM: Un-Methylated promoter; M/UM: both Methylated and Un-methylated promoters.

The data summarized in Table 2 showed that about 87% of breast tumors were methylated in the studied promoter region of the *GBP2* gene, interestingly similar results were observed in the plasma of these patients. In other words, the methylation status of breast tumors was traceable in plasma. The frequency matching of *GBP2* promoter methylation in tumor and plasma samples of breast cancer patients is depicted in Figure 5. In the normal adjacent control group, most of the samples (about 57%) showed the unmethylated region and 40.5% showed both methylated and unmethylated and only 2.4% had *GBP2* promoter methylation. However in normal control group comprised of healthy individuals with no cancer history underwent cosmetic surgery, most of the samples were unmethylated in the studied *GBP2* promoter region. 

**Table 2 -:** Categorization of *GBP2* promoter methylation status.

Sample type	Total number	**Methylated *GBP2* promoter (%)**	**Unmethylated *GBP2* promoter (%)**	**Both methylated and unmethylated *GBP2* promoter (%)**	***P* value *X*** ^*2*^ **test**
BC/plasma	84	74 (88.1%)	8 (9.5%)	2 (2.4%)	*
N/plasma	75	1 (1.3%)	71(94.7%)	3 (4%)	*
BC/tumor	84	73 (86.9%)	8(9.5%)	3 (3.6%)	*
Nadj/tissue	84	2 (2.4%)	48 (57.1%)	34 (40.5%)	*
NC/ tissue	20	1 (5%)	17 (85%)	2 (10%)	*

_BC=Breast cancer, N=Normal, Nadj=Normal adjacent, NC=Normal control. *: X2 test, p ≤ 0.001._

### *GBP2* promoter methylation status in different breast cancer groups based on histopathology situations 

**Figure 6 -:**
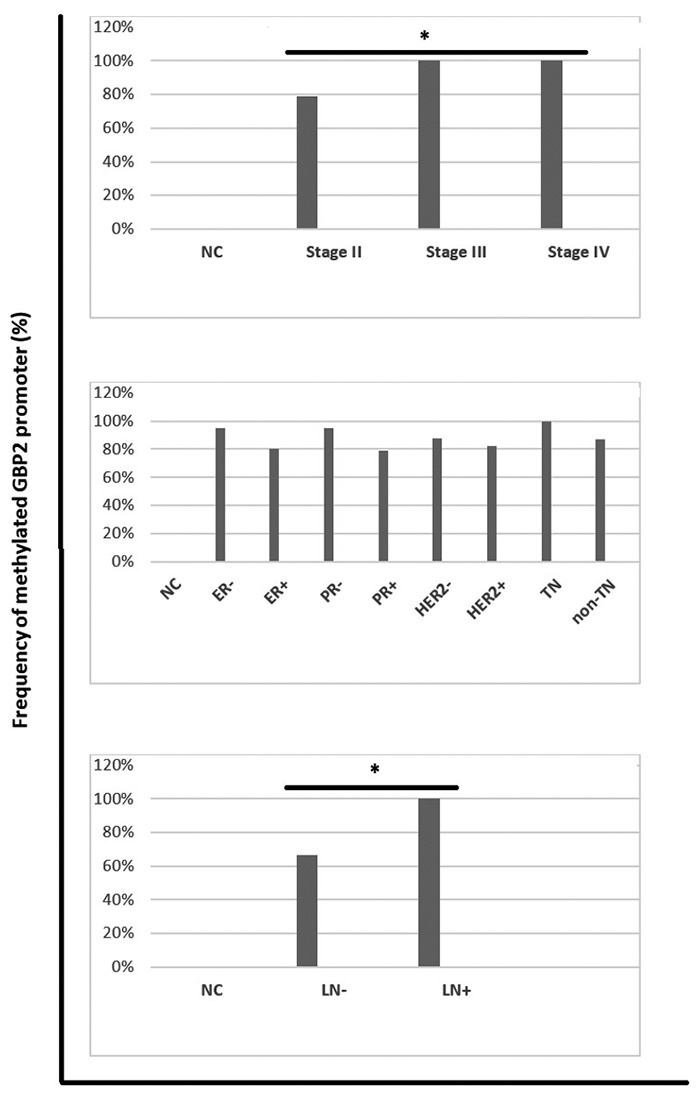
Comparison of methylated *GBP2* promoter frequency in different breast cancer groups based on different stages (up), hormone receptor statues (middle), lymph node involvement (down). ER=estrogen receptor, PR=progesterone receptor, HER2=human epidermal growth factor receptor 2, TN=triple negative, LN=lymph node involvement, NC= normal control. *: *p*<0.01.

Comparison of the studied *GBP2* promoter region methylation patterns in the different subtype of breast cancer group based on lymph node involvement, hormone (estrogen and progesterone) receptors, and HER2 situations and TNM staging has been shown in Figure 6.Our data indicated that stage III and IV, as well as lymph node positive groups with 100% *GBP2* promoter methylation, had significantly higher methylated promoter frequency compared with other studied breast cancer subtypes (*p*<0.001). There were no statistically significant differences in *GBP2* promoter methylation frequency in other mentioned groups in Figure 6.

## Discussion

*GBP2* involves in DNA replication, repair, immune and cytokine signaling pathways. In the various malignancies, such as ovarian cancer, it has been reported as a biomarker (Wang *et al.*, 2016 *). GBP2* may consider as a controlling factor in tumor progression by inhibiting NF-KappaB, Rac and matrix metalloproteinase 9 expressions. This gene has been known to be controlled by p53 (Godoy *et al.*, 2014). An association study on *GBP2* mRNA levels and metastasis-free intervals in 766 node-negative breast carcinoma cases without receiving systemic chemotherapy has shown that higher expression of *GBP2* is correlated with better prognosis in fast proliferating tumors (Godoy *et al.*, 2014).

In esophageal squamous cell carcinoma, up-regulation of GBP2 and IRF-1 as its main transcriptional regulator proposed it as a possible cancer-related marker (Guimaraes *et al.*, 2009).

New findings indicate that *GBP2* regulates mitochondrial fission and suppresses breast cancer invasion by blocking dynamin-related protein 1 (Drp1) translocation from the cytosol to mitochondria. It was reported that Drp1-dependent mitochondrial fission plays a crucial role in breast cancer cell metastasis (Zhang *et al.*, 2017). 

In contrast, studies in murine fibroblasts represent that MuGBP2 expression leads to the reduction in contact growth inhibition and growth factor stimulation dependency. Consequently, these cells grow as the tumor in nude mice (Gorbacheva *et al.*, 2002). According to another study, MuGBP2 protects fibroblasts from paclitaxel-induced apoptosis (Balasubramanian *et al.*, 2011).

Our result revealed a significant reduction in *GBP2* expression in ductal carcinoma breast tissues, compared with normal controls. The lower *GBP2* expression was significantly predominate in triple negative tumors and those with higher stages of the disease and lymph node involvement. It could be concluded that lower expression of *GBP2* may be associated with poor prognosis in breast cancer. This result somehow confirms the *GBP2* tumor suppressor role in breast cancer. In this research, the promoter methylation status of *GBP2* gene, as well as *GBP2* methylation status association with clinicopathologic characteristics were investigated in breast cancer. The results showed that promoter methylation of *GBP2* in tumors of breast cancer patients was significantly associated with some malignant indicators including lymph node involvement, distant metastasis and, higher cancer stages. So the promoter methylation of *GBP2* may predict poor prognosis and invasiveness in breast cancer. The thing was interesting in our study was that about 40% of the normal margin of the tumors called normal adjacent showed the dual pattern of both methylated and unmethylated in their studied *GBP2* promoter regions. Whereas most of the normal breast tissues retrieved from healthy women underwent surgery due to cosmetic purposes showed the unmethylated pattern in their *GBP2* promoter region. We suppose the differences observed in methylation patterns in normal adjacent and normal control may be due to this assumption that normal epithelium surrounding the tumor sites are affected by the signals of neighbor cancerous cells and some of them show the cancerous tumor methylation pattern. We can conclude that in some circumstances normal adjacent tissues may not consider as such an excellent normal control group.

It has been suggested that cfDNA concentration in cancer patients is associated with cancer cells^,^ necrosis, and apoptosis in the tumor microenvironment (Chen *et al.*, 2005). In addition to quantitative changes, cfDNA in tumor cells may possess qualitative changes such as mutations, microsatellite instabilities and methylations (Kasi, 2017; Barault *et al.*, 2018). Follow-up studies reviews confirmed that cancer cells release detectable amounts of cfDNA fragments into biofluids, that bear the unique genetic and epigenetic alterations characterizing the tumor from which they originate (Wan J, 2017). The molecular profiling of cfDNA may serve a potentially useful role in noninvasive cancer management. The origin and molecular properties of cfDNA is a considerable subject. Although a large fraction of cfDNA has been shown to originate from apoptosis, it is becoming clear that cfDNA is released into circulation by multiple mechanisms (Bronkhorst *et al.*2019).

Sizing of cfDNA usually generates a pattern as a “ladder” representing apoptotic fragmentation. The majority of apoptotic DNA fragments has a modal size of ~ 166 bp. However, depending on nuclease action longer DNA fragments could also be produced by apoptosis. The recent studies have reported smaller fragment sizes (as short as 90 bp) for tumor-derived cfDNA compared to wild-type cfDNA. In contrast to apoptosis, cfDNA fragments often observed in cancer patients with an origin from necrosis are larger than 10,000. It may be interesting to note that other forms of cell death such as autophagy, pyroptosis, phagocytosis, mitotic catastrophe, and NETosis, can also serve as sources for cfDNA. In contrast to cellular destruction, a significant fraction of cfDNA is derived from active cellular secretions in the range of 1000-3000 bp, which is not the size typically associated with apoptosis or necrosis. The exact mechanisms involved in the active release of cfDNA remain unclear but it is possible that it would be as a consequence of genomic instability. Moreover, each of these mechanisms are modulated by a wide range of biological and environmental factors (Bronkhorst *et al.*2019).

Gene promoter methylation is a well-known gene expression regulation mechanism. Aberrant gene promoter methylation in cfDNA has been reported as a noninvasive biomarker for detection, differential diagnosis, prognosis and therapy responses in cancers (Warton and Samimi, 2015; Leygo *et al.*, 2017). Here, we investigated *GBP2* promoter methylation status in tumor and normal breast tissues as well as tracing its pattern in plasma sample counterparts. The correlation observed between tumors methylation status and corresponding plasma, certified that plasma *GBP2* methylation pattern could be considered as a representor of the tumor methylation status.

In cancer, cfDNA not only originates from tumor cells but also it originates from cells of the tumor microenvironment, as well as other non-cancer cells. However, to better estimate tumor dynamics, mutation load, progression or assess the efficacy of treatment, the best approach may be to determine the proportion of aberrant *vs*. wild-type DNA, including all forms of cfDNA (Bronkhorst *et al.*2019).

According to the evidence obtained, *GBP2* can be stated as a tumor suppressor gene and its promoter hyper-methylation accompanied by the reduction in its expression. The *GBP2* promoter methylation in circulating DNA may be considered as a possible effective non-invasive molecular marker in poor prognostic breast cancer patients with the evidence of its relation to disease stage and lymph node metastasis. However further studies need to evaluate the involvement of *GBP2* promoter methylation in progression-free survival or overall survival of the patients.
